# Can billing codes accurately identify rapidly progressing stage 3 and stage 4 chronic kidney disease patients: a diagnostic test study

**DOI:** 10.1186/s12882-019-1429-4

**Published:** 2019-07-12

**Authors:** Kabir Jalal, Edwin J. Anand, Rocco Venuto, Joe Eberle, Pradeep Arora

**Affiliations:** 1 Department of Biostatistics, University at Buffalo, State University of New York, 807 Kimball Tower, Buffalo, NY 14214-3000 USA; 2 Division of Nephrology, Department of Medicine, Buffalo, USA; 3 Department of Biomedical Informatics, Buffalo, USA; 4 Intelligent Care Management, Buffalo, USA; 5 Veterans Affairs Medical Center Buffalo, Buffalo, USA

**Keywords:** Progression, CKD, ICD, Sensitivity, Specificity

## Abstract

**Background:**

The International Classification of Diseases (ICD) coding system is the industry standard tool for billing, disease classification, and epidemiology purposes. However, ICD codes are often not assigned or incorrectly given, particularly among Chronic Kidney disease (CKD) patients. Our study evaluated the diagnostic accuracy of CKD-staging ICD codes among CKD patients from a large insurer database in identifying individuals rapidly progressing towards end-stage renal disease (ESRD).

**Patients and methods:**

Serial observations including outpatient serum creatinine measurements collected from 2007 through 2014 of 216,529 patients were examined. The progression of CKD using a serum creatinine based longitudinal mixed-model was contrasted with that documented by CKD-staging ICD codes. Rapid progressors, defined as those with yearly estimated glomerular filtration rate (eGFR) loss greater than 4 ml/min/1.73m^2^) were identified. The diagnosis of CKD using eGFR was also compared to diagnosis using a set of CKD related ICD codes.

**Results:**

Of 10,927 clinically identified CKD patients qualifying for inclusion in the progression analysis, 323 were clinically identified as rapid progressors. CKD-staging ICD codes identified 83 of these, for a sensitivity of 25.7% with positive predictive value (PPV) of 13.74%, and specificity 95.09% with negative predictive value (NPV) of 97.68%. Of 28,762 laboratory-confirmed CKD patients, 9249 had a qualifying ICD code, for a sensitivity of 16% with PPV of 63.10%; Of 187,767 patients with laboratory-confirmed absence of CKD, 182,359 also did not have a qualifying ICD code, for a specificity of 97.12% with NPV of 90.33%.

**Conclusion:**

This study depicts the novel finding that ICD-codes display poor capacity to identify rapidly progressing CKD patients when compared to gold standard eGFR measures, and further demonstrates the limitations of coding in CKD diagnosis. This analysis further defines the limitations of ICD codes in addressing diagnosis of disease severity or disease progression for clinical or epidemiological purposes.

**Electronic supplementary material:**

The online version of this article (10.1186/s12882-019-1429-4) contains supplementary material, which is available to authorized users.

## Background

The International Classification of Diseases (ICD) coding system is the standard tool used by physicians, researchers, insurance providers, and administrators to classify diseases for clinical and epidemiological purposes. Information derived from ICD codes provides the basis of mortality and morbidity statistics that inform the medical community of the burden of disease on the population; such data serves a vital role in determining resource allocation and related medical policy. Insurance providers also use ICD codes as a basis for reimbursement [1].

Consequently, ensuring that reported ICD codes accurately reflect patient diagnoses is of critical importance to the entire medical community.

However, studies designed to examine the agreement of ICD coding with gold-standard clinical markers have shown mixed accuracy of ICD codes depending on disease. Coding accuracy in conditions such as cardiovascular diseases, stroke, or pneumococcal pneumonia is generally accurate, unlike that of Chronic Kidney Disease (CKD) [2–4]. Deficiency of ICD codes in identifying CKD patients and their stage of disease is well described, with other studies reporting low sensitivities and high specificities [5, 6]. These studies, however, are typically based on inpatient data, leaving the more reliable outpatient data insufficiently examined.

The United States Renal Disease System (USRDS) estimates that over thirty-million Americans are affected by CKD, yet fewer than 800,000 have progressed to end stage renal disease (ESRD) [7]. Precise diagnosis of rapidly progressing CKD patients is of critical importance. Such patients benefit from targeted care aimed at maximizing therapy available to delay onset of ESRD. ICD codes are already used to mark CKD staging, and examination of a patient’s coding history may reveal whether the patient is rapidly progressing towards ESRD. However, to date, no attempt to correlate clinically-based measures of progression to ICD-code indicated progression has been made.

This research examines the accuracy of ICD-9-CM coding in relation to progression of CKD using a large third party medical insurer’s claims data, consisting of several years of serial observations on 1.3 million patients. Several CKD associated codes were examined against “gold-standard” clinical markers for diagnosis of CKD to determine if CKD coding remains inadequate in identifying patients.

## Methods

The data analyzed originated from a large third party insurer serving the Western New York and Albany regions of New York State. Comprising of nearly 1.3 million patients over a 10-year period from 2007 through 2017, this database has been used in examining the CKD patients within this sample [8]. Patients with CKD were identified using estimated glomerular filtration rate (eGFR) calculated using the CKD-EPI equation and measured serum creatinine values [9]. Using a unique patient identifier and observation dates, these eGFR values were linked to diagnostic ICD codes where available. ICD-9-CM codes were considered from 2007 through 2014, and ICD-10-CM codes from 2016 through 2017. To avoid potential issues related to transition from ICD-9 to ICD-10, the 2007–2014 and 2016–2017 data was analyzed separately, and the transition year 2015 was excluded.

Patients with serum creatinine measures, age and gender had eGFR values calculated. Due to the absence of racial data, patients were assumed to be white for calculation of eGFR (see discussion of limitations below). Those individuals with two eGFR measures of less than 60 ml/min/1.73m^2^ at least 9 days apart, with no intervening measurement greater than 60 ml/min/1.73m^2^, were identified by their eGFR as stage-3, stage-4, or stage-5 CKD cases, according to interpretation of Kidney Disease Outcomes Quality Initiative (KDOQI) guidelines. Incident and prevalent cases of CKD were included. Note that limited lab values precluded albuminuria-based diagnosis of stage 1 and stage 2 CKD. Individuals with laboratory-confirmed CKD are referred to as eGFR-CKD.

ICD-9-CM codes were used to identify patients who were diagnosed with CKD. The following code groups were considered: Chronic Renal Failure (403.11, 404.12, 404.13, 404.92, 404.92, 585, 585.1, 585.2, 585.3, 585.4, 585.5, 585.6, 585.9, 586, 587), Diabetic nephropathy (250.4, 250.40, 250.41), and systemic disease causing CKD (203.0, 277.3, 287.0, 446.0, 446.2, 446.4, 446.6, 710.0, 710.1). ICD-10-CM codes for chronic kidney disease (N18.1, N18.2, N18.3, N18.4, N18.5, N18.6, N18.9) were considered. Patients with at least one occurrence of any listed code were considered ICD-CKD. Thus, the total sample includes patients with and without laboratory-confirmed CKD, as well as patients with and without ICD-CKD.

Among eGFR-CKD patients, a longitudinal mixed model analysis was used to model eGFR over time [10]. Patients who experienced a yearly loss of eGFR of greater than 4 ml/min/1.73m^2^ were considered rapid progressors [11, 12]. Clinical evidence of onset of CKD-stage 3 or stage 4 was considered baseline, and patient observations were included until CKD-stage 5 or ESRD treatment initiation. Patients not having at least 3 years of follow-up data or five observations were excluded from analysis. Time, measured in quarter-year increments, was included as a fixed effect with a random intercept and a random time effect additionally was included.

From this model, Estimated Best Linear Unbiased Predictors (EBLUPs) were categorized according to their slope into those who experience rapid progression and those who do not [13]. An individual was determined to be a rapid progressor by ICD-code if the patient had at least two staging codes (585.3, 585.4, 585.5) that indicated increasing disease severity. Thus, each patient in the progression analysis was assigned an eGFR (eGFR-progressor) and ICD (ICD-progressor) indicator of being a rapid progressor or not.

Descriptive statistics regarding the portion of the sample showing signs of comorbidities, as well as gender and age > 65 were generated for the overall sample, eGFR-CKD sample, the ICD-CKD sample, as well as the sample considered for progression analysis.

Assessment was made of the accuracy of ICD-CKD to indicate eGFR-CKD with estimates and 95% confidence intervals for sensitivity (#true positives/[#true positives + #false negatives]), specificity (#true negatives/[#true negatives + #false positives]), positive predictive value (PPV; #true positives/[#true positives + #false positives]) and negative predictive value (NPV; #true negatives/[#true negatives + #false negatives]) rates. These quantities are collectively referred to as “performance measures” throughout this manuscript.

ICD-code derived CKD diagnosis performance measures were contrasted in four different ways with performance measures determined from the “gold-standard” eGFR-based CKD definition.

- Progression Analysis: Presence (or absence) of two CKD staging/ESRD codes (585.3, 585.4, 585.5, 585.6) indicating increasing severity was compared to model-identified rapid progressors.
- Overall Analysis: Presence (or absence) of any qualifying code was compared to the clinical diagnosis results based on eGFR-CKD guidelines for an overall measure of accuracy. This analysis was repeated using data from 2016 and 2017 to assess overall ICD-10 coding accuracy.
- Stage-Stratified Analysis: Presence (or absence) of any qualifying code was compared against patients at their highest eGFR based stages of CKD (stage 3–5) for a ‘stage-stratified’ comparison to determine if ICD-diagnosis rates improve with advancing clinical stage.
- ROC Analysis: Agreement of ICD- and eGFR-CKD diagnoses was modeled against gender, age > 65, and comorbid conditions (proteinuria, diabetes, congestive heart failure, other heart diseases, and hypertension) in a multivariate logistic regression. Receiver operating characteristic (ROC) curves were generated using the Mann-Whitney association to estimate the area under the curve (AUC). A non-informative curve with AUC of 0.5 was held as reference, and every other curve was compared using a non-parametric approach [14].

## Results

Of the approximately 1.3 million patients in the insurance database, 216,529 qualified for analysis. From the 216,529 qualifying patients, 28,762 were identified as eGFR-CKD and 14,657 were identified as ICD-CKD. Twenty-six thousand three hundred eleven patients showed clinical signs of stage 3; 2007 stage 4; and 444 stage 5. Ultimately, 23,802 reached a maximum of stage 3; 4234 stage 4; and 726 stage 5.

Ten thousand nine hundred twenty-seven of the 28,762 eGFR-CKD patients and 4650 of the 14,657 ICD-CKD met inclusion criteria for the progression analysis model. These included 2891 incident and 8036 prevalent cases. At baseline, 10,339 patients were in stage 3, and 588 were in stage 4. By the study end, 7917 were in stage 3, 1248 in stage 4, while 1537 patients ended with an eGFR above 60, and 225 had reached stage 5. The mean time in the database prior to meeting eGFR-CKD criteria among incident patients was 765 days.

**Fig. 1:**
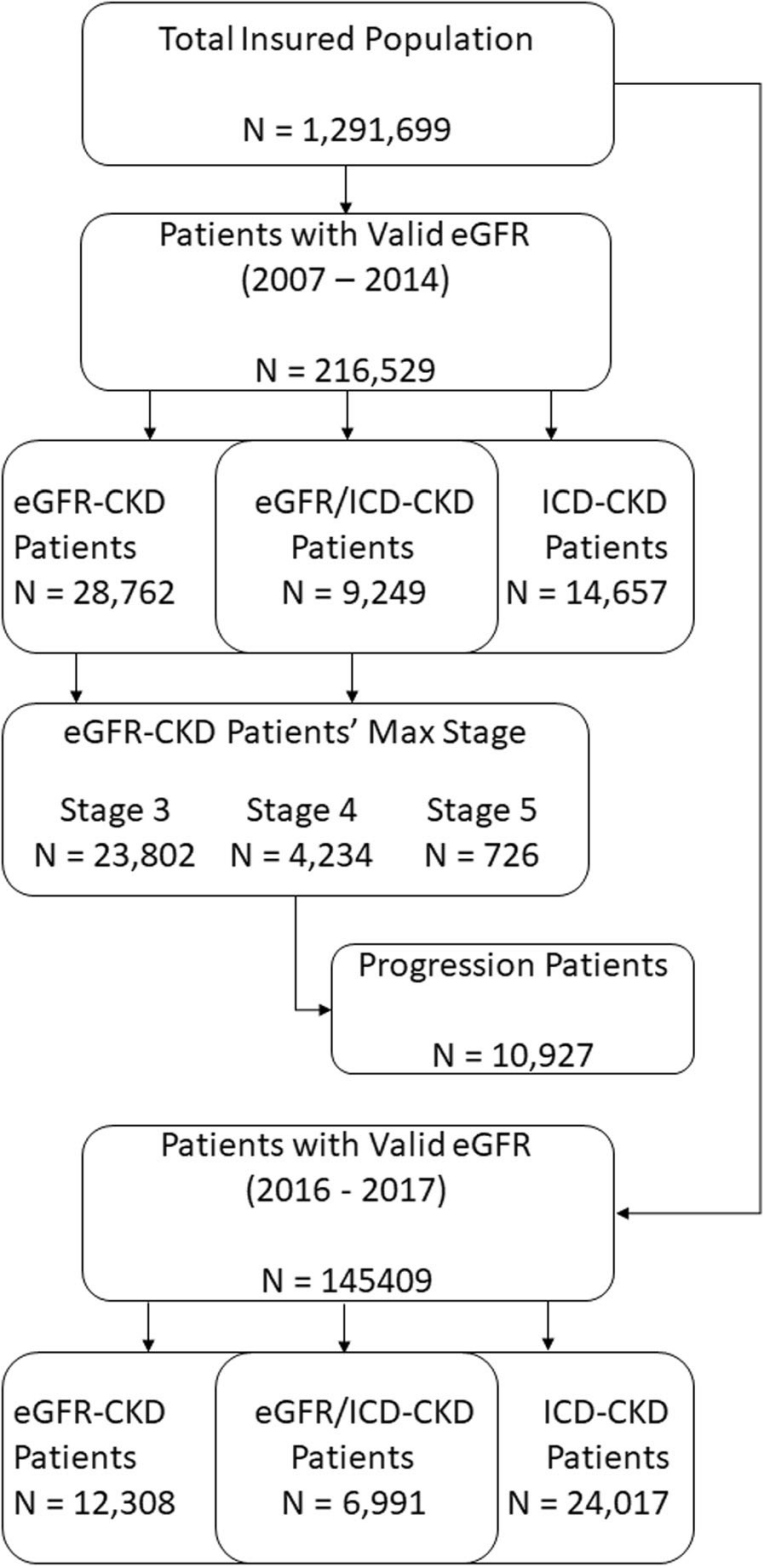
Sample inclusion/exclusion summary. Patients were selected from HealthNow insurance database and required to have non-missing estimated glomerular filtration rate (eGFR) for inclusion

Figure 1 summarizes patient inclusion rates. A complete population breakdown, with details regarding patient demographics and comorbid conditions, is available in Table 1. Results of McNemar’s test showed differences in proportions across all groups (*p* < 0.0001).

**Table 1:** Demographic Summary

Variable	Progression Sample (*N* = 10,927)	eGFR- Progressors (*N* = 323)	ICD Progressors (*N* = 604)
	% Yes	% Yes	% Yes
Male Gender	39.21	51.70	50.00
Age > 65	76.57	71.52	69.37
Proteinuria	4.36	17.65	21.52
Diabetes	42.00	67.49	56.46
Hypertension	88.89	93.19	92.55
Congestive Heart Failure	12.87	21.98	16.23
Other Heart Issues	56.31	64.09	58.28
	Overall Sample (*N* = 216,529)	eGFR-CKD (*N* = 28,762)	ICD-CKD (*N* = 14,657)
	% Yes	% Yes	% Yes
Male Gender	45.80	41.40	41.25
Age > 65	27.53	76.57	57.62
Proteinuria	1.040	7.840	12.53
Diabetes	31.40	50.00	49.37
Hypertension	74.04	93.96	89.32
Congestive Heart Failure	9.440	31.69	31.31
Other Heart Issues	68.68	80.85	83.88

Of the 10,927 patients considered in the progression analysis, 323 were identified as rapid progressors. Although 626 were observed to have multiple ICD codes suggesting disease progression, only 89 of these patients were among the 323 rapid progressors. Sensitivity was 25.7% (21.02, 30.83) with PPV 14.22% (13.74, 11.10), and specificity of 94.94% (94.66, 95.49) with NPV of 97.73% (97.37, 97.96). Table 2 summarizes the progression analysis sample.

**Table 2:** Contingency Table of eGFR-based identification against ICD Identification of Rapid Progressors

		eGFR-Progressors		Total
		Yes	No	
ICD-Progressors	Yes	89	537	626
		0.81%	4.91%	5.73%
	No	234	10067	10301
		2.14%	92.13%	94.27%
	Total	323	10604	10927
		2.95%	97.04%	100%

**Fig. 2:**
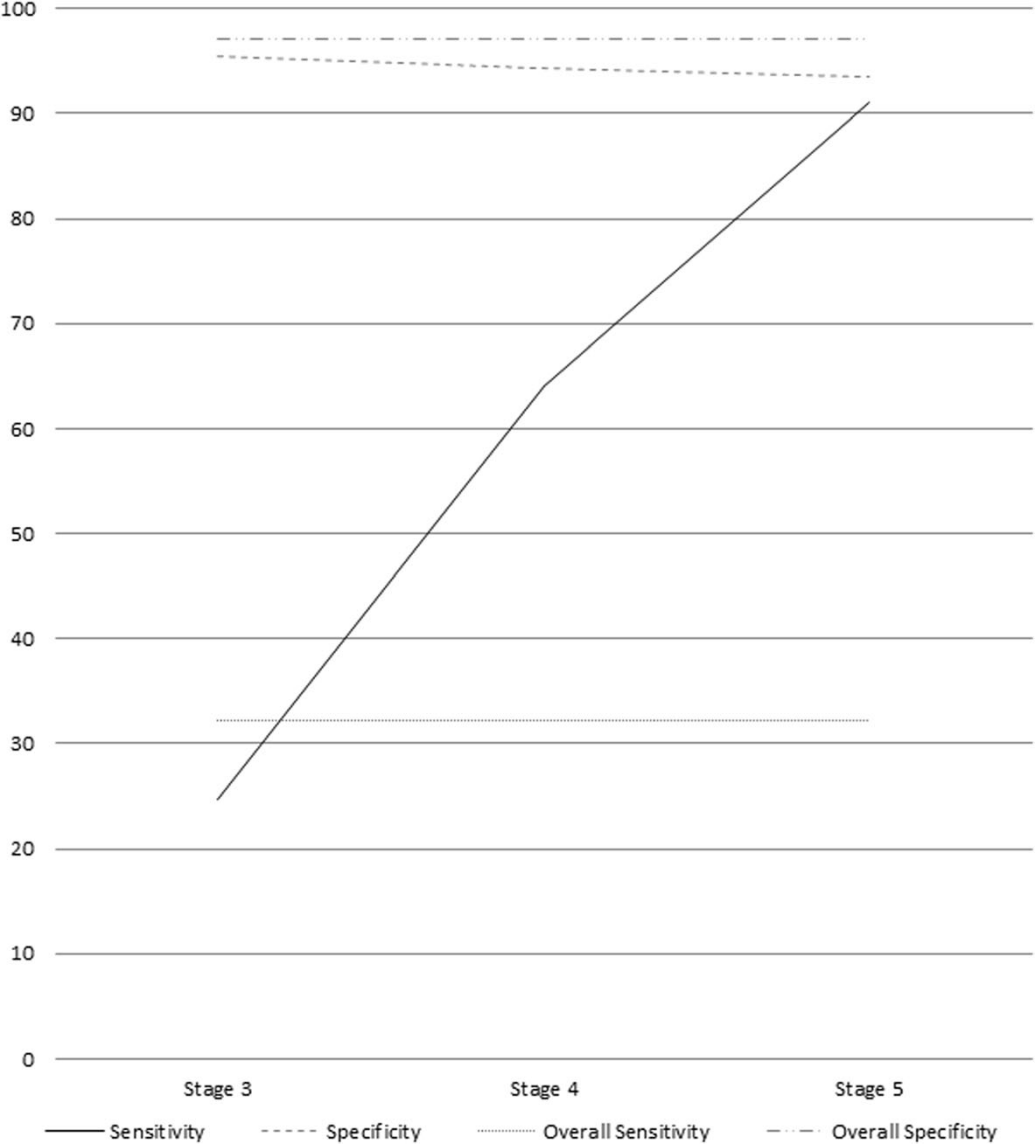
Overall and Stage-specific Sensitivity and Specificity Rates. Sensitivity and specificity rates for chronic kidney disease (CKD) diagnosis using International Classification of Diseases (ICD) codes against gold-standard Kidney Disease Outcomes Quality Initiative (KDOQI) guidelines

For the 2007–2014 sample, ICD codes correctly identified 9249 of 28,762 overall eGFR-CKD patients; 5205 of 23,802 stage-3 eGFR-CKD patients; 1437 of 4234 stage-4 eGFR-CKD patients; 342 of 726 stage-5 eGFR-CKD patients. In the overall analysis, sensitivity was 32.16% (95% CI: 31.62, 32.70) with 63.10% (95% CI: 62.32, 63.88) PPV and specificity of 97.12% (95% CI: 97.04, 97.20) with NPV of 90.33% (95% CI: 90.20, 90.46), respectively. The sensitivity and NPV increased from 24.68% (95% CI: 24.13, 25.23) and 91.12% (95% CI: 90.99, 91.24) among patients showing clinical evidence of stage-3 eGFR-CKD to 91.05% (95% CI: 88.73, 93.02) and 99.97% (95% CI: 99.96, 99.98) among stage-5 patients. The overall and stage-specific results are summarized in Fig. 2. Complete results with 95% confidence intervals are available in Additional file 1.

**Fig. 3:**
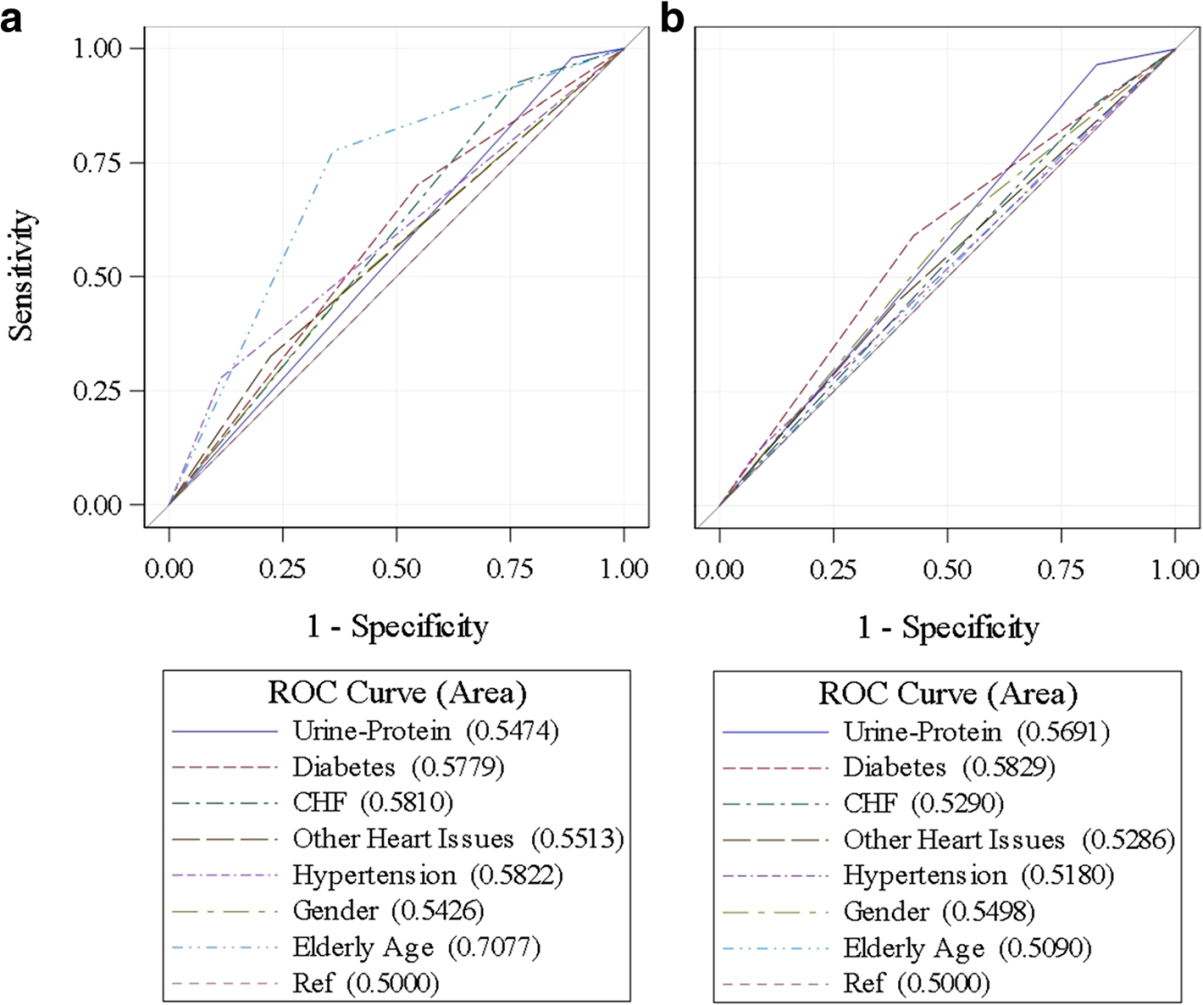
Receiver Operating Characteristic (ROC) Curves. **a** ROC curves for chronic kidney disease (CKD) diagnosis using International Classification of Diseases (ICD) codes against gold-standard Kidney Disease Outcomes Quality Initiative (KDOQI) guidelines. Diagnostic accuracy only improves with increasing age. **b** ROC curves for identifying rapidly-progressing CKD patients using ICD codes against gold standard KDOQI guidelines. Diagnostic accuracy shows no clinically meaningful improvement regardless of subgroup

In identifying rapid progressors, ROC analysis showed that codes offered no improvement in discriminating rapid progressors over an arbitrary decision when controlling for comorbidities. In identifying the general CKD sample, only age over 65 offered a significant improvement in discriminating CKD, with the only clinically predictive AUC (AUC = 0.7077, 95% CI: 0.70, 0.71) among variables considered. Figure 3 depicts the curves. 95% intervals and associated *p*-values are available in Additional file 1.

The 2016 and 2017 sample consisting of ICD-10 codes had 145,409 total patients having valid eGFR, with 12,308 eGFR-CKD patients and 24,017 ICD-CKD patients. Sensitivity rates derived from the 2016–17 data were 29.11% (95% CI: 28.53, 29.68), with PPV of 56.80% (95% CI: 55.93, 57.68) and specificity of 95.62 (95% CI: 95.50, 95.74) with NPV of 87.21% (95% CI: 87.03, 87.39). Aside from a marked increase in the number of patients with ICD codes, these figures do not differ substantially from the ICD-9 derived values presented in this analysis. Detailed results are available in Additional file 1.

## Discussion

The ability to detect which individuals in a large CKD database whose renal function is on an accelerated downward trajectory is the so-called “Holy Grail” of CKD analytics. This allows for programmatic approaches to interventions designed to slow the disease course or provide expeditious care for advanced CKD. We are the first to report on the performance of ICD codes in CKD progression. Our data showed that coding is particularly inaccurate in identifying CKD patients who were rapidly deteriorating, with a sensitivity of 25.7% compared to 32% in identifying CKD in general. Not unexpectedly, sensitivity increased from 25, 64 and 91% from stage 3, stage 4, and stage 5 CKD respectively.

This progression model identified 323 of 10,927 patients with a yearly loss of eGFR greater than 4 ml/min/1.73m^2^. Of these 323 eGFR-CKD rapid progressors, 176 did not experience a loss of eGFR sufficient to warrant a change in ICD-stage classification. However, of those 176 there were 128 patients with eGFR indicative of ICD-stage 3 CKD. Taken together, this strongly suggests that additional CKD-related codes would likely help in identification of rapid progressors. Indeed, current codes do not have the level of granularity to mark small changes in eGFR.

The rate of rapid progressors found in our sample was approximately 0.029%. This is nearly double than a rate of 0.016% found in a 2016 VA study [12]. However, that study focused on elderly patients, who experience a slower rate of progression than the general adult population we have examined here. Given that population difference, it is reasonable to assume that results of this study are consistent with previous findings.

Our results showed much lower sensitivity in detecting stage 3 CKD by codes than reported in most of prior studies. Selected studies are summarized in Table 3. A meta-analysis of 25 studies showed a median sensitivity of 41%, ranging from 3 to 88%, and a PPV of 78%, ranging from 29 to 100% [5]. A similar review of 30 studies showed a wide varying sensitivity (8–83%), but with specificity values above 90% [15]. A study of two practice-based research networks examining ICD-9 codes regarding chronic kidney disease (CKD) showed 47% of patients with a CKD ICD-9 code did not qualify for a diagnosis of CKD [4]. A population-based study of elderly patients employed an algorithm utilizing ICD codes, providing a sensitivity of 18% and PPV of 85% among patients with eGFR less than 60, and a sensitivity of 58% with a PPV of 32% when eGFR was less than 30 [6]. (Table 3).

**Table 3:** Characteristics of Studies on Diagnostic Accuracy of Chronic Kidney Disease

Reference	Location	Population selection criteria	Study timeframe	Sample size	Gold-standard definition of kidney disease	Diagnostic tool for kidney disease	Sensitivity & specificity	Additional notes
*Current Study*	Western New York	Outpatient data with two valid serum creatinine	2007–2014	216,529	KDOQI based on CKD-EPI eGFR	27 ICD-9 Codes	*See Paper*	Gold-Standard based on 2 eGFR measures
Chase et al. 2010 [16]	Columbia University Medical Center	Outpatient data with two elevated serum creatinine values	2003–2006	175	KDOQI based on CKD-MDRD eGFR	Electronic Health Records containing CKD documented in notes	95.4–99.8 & 99.8	All hypertensive patients
Ronksley 2012 [17]	Alberta, Canada	Outpatient with two elevated serum creatinine values	2004–2005	321,293	KDOQI based on CKD-MDRD eGFR	25 ICD-9 Codes	18.9–29.3 & 94.6–98.5	Gold-Standard based on 2 eGFR measures
Cipparone 2015 [4]	Buffalo, Kansas	Inpatient Chart Review	–	325	Chart review protocol based on KDOQI Guidelines	ICD-9585.3 Code	–	Prevalance of misdiagnosis; no Sensitivity or Specificity
Fleet 2013 [6]	Ontario, Canada	Outpatient age > 65	2007–2010	123,499	CKD-EPI eGFR < 60; < 45; < 30	Algorithm of hospital encounter and 11 ICD-9 Codes	18 & 98.2	Gold-Standard based on only 1 eGFR measure
Winkelmayer 2005 [24]	Pennsylvania	Medicare Inpatients	1999–2000	1852	CKD-MDRD eGFR < 60	22 ICD-9 Codes	2–27 & 93–100	Gold-Standard based on only 1 eGFR measure
Kern 2006 [22]	US VA and Medicare Systems	Inpatient and Outpatient Diabetics in VA System	1999–2000	263,730	CKD-MDRD eGFR < 60	79 ICD-9 Codes	20–41 & 95–99	Gold-Standard based on only 1 eGFR measure
Stevens 2005 [18]	Laboratory Corporation of America, Columbus, OH	Outpatient age > 39	2002–2003	277,111	CKD-MDRD eGFR < 60	51 ICD-9 Codes	10–51 & 95–98	Gold-Standard based on only 1 eGFR measure
Navaneethan 2011 [19]	Cleveland Clinic Patients	Outpatient with two elevated serum creatinine values and/or two ICD-9 diagnoses	2005–2010	296,249	KDOQI based on CKD-MDRD eGFR	8 ICD-9 Codes	> 80	Gold-Standard based on 2 eGFR measures
Lardon 2015 [20]	French PMSI Hospitals	Inpatient age 12–65 or 80	January, 2014	533	eGFR	Drools rules engine based on EHR and ICD-10	–	Analyzed hospital stays, rather than patients

The reason for this discrepancy could be the clinical population studied. Specifically, as most US based studies collected data from hospitalized patients. Inpatient labs are volatile in nature and their inclusion could result in an increase of patients with AKI. These studies also sometimes focused on patients presenting with a particular comorbidity, such as diabetes, congestive heart failure, or hypertension, potentially excluding CKD patients without another presenting condition. In contrast, the vast majority of our data is derived from more stable outpatient labs and included all patients covered by a single insurer. Together with the longitudinal nature of our data and the inclusion criteria (minimum of five observations and 3 years of follow-up) placed on the sample for progression analysis limit any potential contamination with AKI patients.

Our research supports findings from previous studies while offering additional information regarding advanced CKD stages 4 and 5. For example, a study using outpatient labs with a similarly large sample size based in Alberta, Canada showed the predictive characteristics of billing codes were similar with a PPV in the range of 60% [17]. As demonstrated in our data, they also found billing codes were more reliable when the estimated GFR was less than 30 ml/min. The PPV was 85% for patients with eGFR of less than 60 ml/min, but decreased to 32% for eGFR < 30 ml/min. An Ontario, Canada based study utilized a combination of physician billing codes and hospitalization codes, showed sensitivity in the range of 20% with PPV of about 60% overall [6]. In this study a combination of 9 physician codes and 2 inpatient codes that referred to CKD were used. Our results confirm the observed trend of more advanced stages of CKD have increasing sensitivity and NPV. However, decreasing specificity and poor PPV preclude any notion that coding is substantially improved in advanced stages. This seemingly contradictory pattern is a consequence of a larger proportion of false negatives than false positives as disease severity increases.

When assessing other diseases, ICD codes generally have much better performance against gold-standard diagnostic tools. A single-hospital study of ICD-9-CM codes in detecting pneumococcal pneumonia showed a 58.3% sensitivity rate [2]. A 12-year retrospective study in an Australian population reported sensitivity and specificity for various cardiovascular diseases, from 74.2% sensitivity and 97.6% specificity for coronary heart disease to 38.7 and 99.9% for ischemic stroke [21]. The difference between the accuracy of coding patients with cardiovascular diseases, stroke, pneumococcal pneumonia and CKD is likely at least in part to be a consequence of the primacy of their life-threatening acute, obvious, and readily documentable conditions. This is in contrast to the indolent and often secondary or even tertiary diagnosis of especially the moderate stages of CKD. The subtle nature of CKD therefore may cause primary care and other physicians to code other, more obvious diseases first, despite similar prevalence rates. Patients may not yet be referred to nephrologists who would likely be more accurate with their codes than a primary care physician for that same reason.

This study has potential limitations, chief among them the lack of racial data. Consequently, eGFR was based on the assumption that all patients were white, leading to decreased eGFR readings on average. However, historical demographic data of the region show that only 13% of the region’s population is African American, which would limit bias in the performance measures [23]. This could partially explain the generally worse coding accuracy observed in our data.

Another limitation is that our study used exclusively ICD-9 codes, and the recent transition to ICD-10 may have some effect on these results. However, the analysis of the 2016–17 data using ICD-10 codes failed to reveal any substantial discrepancies between the two coding revisions with respect to accuracy of identifying CKD.

The lack of longitudinal data and relatively smaller sample, however, precluded a progression or stage-stratified analysis. As additional years of longitudinal data accumulate, the capability of ICD-10 codes to identify rapid progressors will be evaluated. However, the results are unlikely to change as there are no ICD codes even in the revised version (ICD-10-CM) to capture rapid progression of CKD. Indeed, specific CKD-related codes are mapped one-to-one between ICD-9 and ICD-10. As noted above, neither are codes available to identify minor changes in eGFR. For example, a patient with CKD stage 3 (ICD-9 code: 585.3) and a starting eGFR of 55 ml/min, would need to lose another 25 ml/min of eGFR to a new value of 30 ml/min to be reclassified as CKD stage 4 (ICD-9: 585.4). In any case, separation of ICD-9 code 585.3 (eGFR 30–59) into distinct stage 3a (eGFR 44–59) and stage 3b (eGFR 30–44) codes and/or inclusion of a code indicating rapid progression in a subsequent ICD revision may ease identification of this critical population.

## Conclusion

In summary this data, from a single database composed of over 1.3 million patients followed for as long as 8 years (2007–2015), shows that the inaccuracy of administrative codes in capturing eGFR-CKD extends to indicators of disease progression. This splay between the effectiveness of administrative codes versus clinically derived data precludes the discovery of disease course and behavior as noted in the clinical progression analysis discussed above, and we conclude that administrative codes cannot be effectively used to identify CKD patients, rapidly progressing or otherwise. Future work will include further refining the progression model to include covariates, highlighting risk factors to identify rapidly progressing patients.

## Additional file

Additional file 1: **Tables S1.** Contains additional supporting tables not directly relevant to the manuscript. (XLSX 15 kb)

## Data Availability

The data that supported the findings of this study are available from the University at Buffalo’s Institute for Healthcare Informatics (IHI), but restrictions apply to the availability of these data, which are used under license for the current study and so are not publicly available. Data may be available from the authors upon request and consent of the IHI. Inquiries should be directed to IHIreq@buffalo.edu.

## Abbreviations

AUC: Area Under the Curve
CKD: Chronic Kidney Disease
eGFR: estimated Glomerular Filtration Rate
ESRD: End-Stage Renal Disease
ICD: International Classification of Diseases
KDOQI: Kidney Disease Outcomes Quality Initiative
NPV: Negative Predictive Value
PPV: Positive Predictive Value
ROC: Receiver Operator Characteristic

## References

[1] O’Malley KJ, Cook KF, Price MD (2005). Measuring diagnoses: ICD code accuracy. Health Serv Res.

[2] Guevara RE, Butler JC, Marston BJ (1999). Accuracy of ICD-9-CM codes in detecting community-acquired pneumococcal pneumonia for incidence and vaccine efficacy studies. Am J Epidemiol.

[3] Goldstein LB (1998). Accuracy of ICD-9-CM coding for the identification of patients with acute ischemic stroke: effect of modifier codes. Stroke.

[4] Cipparone CW, Withiam-Leitch M, Kimminau KS (2015). Inaccuracy of ICD-9 codes for chronic kidney disease: a study from two practice-based research networks (PBRNs). J Am Board Fam Med.

[5] Vlasschaert ME, Bejaimal SA, Hackam DG (2011). Validity of administrative database coding for kidney disease: a systematic review. Am J Kidney Dis.

[6] Fleet JL, Dixon SN, Shariff SZ (2013). Detecting chronic kidney disease in population-based administrative databases using an algorithm of hospital encounter and physician claim codes. BMC Nephrol.

[7] United States Renal Data System (2017). 2017 USRDS annual data report: epidemiology of kidney disease in the United States.

[8] Arora P, Elkin PL, Eberle J (2015). An observational study of the quality of care for chronic kidney disease: a Buffalo and Albany, New York metropolitan area study. BMC Nephrol.

[9] Levey AS, Stevens LA, Schmid CH (2009). A new equation to estimate glomerular filtration rate. Ann Intern Med.

[10] Laird NM, Ware JH (1982). Random-effects models for longitudinal data. Biometrics.

[11] Go AS, Yang J, Tan TC (2018). Contermporary rates and predictors of fast progression of chronic kidney disease in adults with and without diabetes mellitus. BMC Nephrol.

[12] Arora P, Jalal K, Gupta A (2017). Progression of kidney disease in elderly stage 3 and 4 chronic kidney disease patients. Int Urol Nephrol.

[13] Robinson GK (1991). That BLUP is a good thing: the estimation of random effects. Stat Sci.

[14] DeLong ER, DeLong DM, Clarke-Pearson DL (1988). Comparing the areas under two or more correlated receiver operating characteristic curves: a nonparametric approach. Biometrics.

[15] Grams ME, Plantinga LC, Hedgeman E (2011). Validation of CKD and related conditions in existing data sets: a systematic review. Am J Kidney Dis.

[16] Chase HS, Radhakrishnan J, Shirazian S (2010). Under-documentation of chronic kidney disease in the electronic health record in outpatients. J Am Med Inform Assoc.

[17] Ronksley PE, Tonelli M, Quan H (2012). Validating a case definition for chronic kidney disease using administrative data. Nephrol Dial Transplant.

[18] Stevens LA, Fares G, Fleming J (2005). Low rates of testing and diagnostic codes usage in a commercial clinical laboratory: evidence for lack of physician awareness of chronic kidney disease. J Am Soc Nephrol.

[19] Navaneethan SD, Jolly SE, Schold JD (2011). Development and validation of an electronic health record-based chronic kidney disease registry. Clin J Am Soc Nephrol.

[20] Lardon J, Asfari H, Souvignet J (2015). Improvement of diagnosis coding by Analysing EHR and using rule engine: application to the chronic kidney disease. Stud Health Technol Inform.

[21] Harriss LR, Ajani AE, Hunt D (2011). Accuracy of national mortality codes in identifying adjudicated cardiovascular deaths. Aust N Z J Public Health.

[22] Kern EF, Maney M, Miller DR, et al. Failure of ICD-9-CM codes to identify patients with comorbid chronic kidney disease in diabetes. Health Serv Res. 2006;41(2):564–580. 10.1111/j.1475-6773.2005.00482.x 10.1111/j.1475-6773.2005.00482.x PMC1702507 16584465

[23] United States Census Bureau / American FactFinder. “DP-1: Race.” *2010 Census*.U.S. Census Bureau, 2010. Available from: https://factfinder.census.gov/faces/nav/jsf/pages/index.xhtml. Accessed 14 Apr 2018.

[24] Winkelmayer WC, Schneeweiss S, Mogun H, Patrick AR, Avorn J, Solomon DH. Identification of individuals with CKD from Medicare claims data: a validation study. Am J Kidney Dis. 2005;46(2):225–32. 10.1053/j.ajkd.2005.04.029 16112040

